# Association of childhood trauma, social support, cognition, and suicidality in females with bipolar disorder

**DOI:** 10.1186/s12888-024-05672-9

**Published:** 2024-04-02

**Authors:** Min Yang, Jiaxin Li, Yaqian Fu, Guotao Wang, Minghui Liu, Jindong Chen, Jieyu Liu

**Affiliations:** 1 Department of Psychiatry, National Clinical Research Center for Mental Disorders and National Center for Mental Disorders, The Second Xiangya Hospital of Central South University, 410011 Changsha, Hunan China; 2 Department of Ultrasound Diagnosis, The Second Xiangya Hospital of Central South University, 410011 Changsha, Hunan China

**Keywords:** Bipolar disorder, Female, Childhood trauma, Social support, Cognition, Suicidality

## Abstract

**Background:**

Bipolar disorder (BD) is a severe mental disorder with heavy disease burden. Females with BD are special populations who suffer a lot from childhood trauma, social support, cognitive deficits, and suicidality. In this study, the relationship among childhood trauma, social support, and clinical symptoms of BD was investigated and the risk factors for suicidality were explored in female patients with BD.

**Methods:**

This study included 57 drug-naive female BD patients, 64 female BD patients with long-term medication, and 50 age-matched female healthy controls. Childhood trauma, social support, clinical symptoms, cognition, and suicidality (suicide ideation, suicide plan, suicide attempt, suicide frequency) were measured with scales.

**Results:**

Compared with healthy controls, females with BD showed higher levels of childhood trauma and suicidality, and lower levels of social support and cognitive deficits. In the drug-naïve BD group, social support mediated the relationship between childhood trauma and insomnia symptoms (indirect effect: ab = 0.025). In the BD with long-term medication group, mania symptom was associated with suicide plan (OR = 1.127, *p =* 0.030), childhood trauma was associated with suicide attempt (OR = 1.088, *p =* 0.018), and years of education (OR = 0.773, *p =* 0.028), childhood trauma (OR = 1.059, *p =* 0.009), and delayed memory (OR= 1.091, p= 0.016) was associated with suicide frequency (OR = 1.091, *p =* 0.016).

**Conclusions:**

This study provides initial evidence that social support partially explains the relationship between childhood trauma and clinical symptoms in females with BD. Additionally, mania symptoms, childhood trauma, and delayed memory were risk factors for suicidality. Interventions providing social support and improving cognitive function may be beneficial for females with BD who are exposed to childhood trauma and with high suicide risk.

## Introduction

Bipolar disorder (BD) is a severe mental disorder characterized by recurrent mood episodes, including depressive and manic/hypomanic episodes [1]. Patients with BD have low-quality lives, such as comorbidities [2], impaired psychosocial functioning [3], low employment [4], and suicide ideation and suicide attempts [5]. Females with BD are important and special populations who may need more attention. Partial S reviewed that females with BD are characterized by late age of onset, seasonality, atypical presentation, high degree of mixed episodes, and a high frequency of medical and psychiatric co-morbidity [6]. In addition, women shoulder the burden of bearing children, and the reproductive period of life is strongly influenced by BD [6]. Moreover, a recently published study reported that females with BD were overlooked by their mothers during their upbringing [7]. All these factors may aggravate the disease condition and make treatment extremely challenging in females with BD.

Early-life stress, including childhood trauma, can disturb the development of the brain and thus increase the risk of mental illness [8]. It was found that BD patients with childhood trauma showed greater symptom severity [9], and increased risk of nonsuicidal self-injury [10]. Wrobel et al. reported that depressive symptoms in BD patients were positively associated with childhood trauma [11]. A meta-analysis of Agnew-Blais et al. also reported that depressive, manic, and psychotic symptoms of BD were all positively associated with childhood trauma, and the number of depressive and manic episodes was also positively associated with childhood trauma [12]. Colic et al. found that females with BD experienced more severe childhood trauma than males with BD [13]. Although researchers have recognized the role of childhood trauma in bipolar disorder, most studies were conducted in BD patients with medication, and there is a lack of literature comparing self-report childhood trauma between drug-naïve BD patients and those with medication.

Social support is a protective factor for mental health. Studies have shown that low social support worsens the outcome of BD and causes a high caregiver burden [14]. Some researchers proposed that fully understanding the role of social support in BD could provide information on prevention and treatment [15]. Compared with males, females were more sensitive to low social support [16] and tended to seek and obtain social support when faced with environmental stressors [17]. Therefore, females with BD may benefit more from social support interventions [18]. Additionally, some studies have found that social support was associated with mood symptoms and could mediate the negative impact of childhood trauma on mood symptoms [19, 20]. However, there is a gap in the understanding of the interaction between childhood trauma and social support in drug-naïve patients with BD and comparing this interaction between drug-naïve BD patients and BD patients with medication.

Cognitive deficits of BD patients were widely reported in a range of domains, including attention, verbal learning, and mental flexibility [21]. Zhang et al. reviewed that cognitive dysfunction is one important parameter implicated in suicidality [22], but Gilbert et al. did not find the association between cognition and suicide attempt [23]. Xu et al. reported that there was a difference in domains of cognition between male and female BD patients [24]. And there may be different neurobiological mechanisms underlying suicide ideation and suicide attempt [22]. Considering gender and different categories of suicidality in the relationship between cognitive deficits and suicidality may make development in the treatment and prevention of suicidality.

It was reviewed that the annual suicide rate in BD was approximately 0.9% [25]. One study found that BD patients with suicide attempts were more likely to be females [26]. More importantly, Rowe et al. reported that both childhood trauma and social support served as predictors of suicide ideation [20]. However, few studies explored childhood trauma, social support, and suicidality independently in female patients. Identifying the risk factors of suicidality for females with BD may help improve clinical outcomes.

In this study, childhood trauma, social support, cognition, and suicidality were compared in drug-naïve female BD and BD patients with long-term medication. Furthermore, the role of social support in the relationship between childhood trauma and clinical symptoms were explored. More importantly, the predictors of suicidality including suicide ideation, suicide plan, suicide attempt, and suicide frequency were explored.

## Methods

### Participants

A total of 121 female patients with BD were recruited from the Department of Psychiatry of Second Xiangya Hospital of Central South University, Changsha, China. The patients were divided into two patient groups: the drug naïve BD group and BD with long-term medication. The inclusion criteria for BD patients were: (1) aged 15–50 years, female; (2) meet the diagnostic criteria of the Diagnostic and Statistical Manual of Mental Disorders, Fifth Edition (DSM-5), and diagnosed with BD by at least one psychiatrist at or above the deputy director level using MINI-International Neuropsychiatric Interview Mini diagnostic interview (M.I.N.I); (3) willing to participate and signed informed consent. Additionally, the drug naïve BD group should not have use of previous and current psychotropic drugs, and the BD with long-term medication group should have a long-term medication for at least 6 months. Fifty-three female healthy controls (HCs) were recruited from nearby communities and universities by advertisements and screened for psychiatric disorders. The participants of the HC group should have never been diagnosed with any psychiatric disorder and should not have a family history of psychiatric disorders. Three of the 53 HCs were excluded because they had a family history of psychiatric disorders. The exclusion criteria for all participants were: (1) a history of craniocerebral trauma or diagnosed with neurological or organic mental diseases; (2) complicated with other psychiatric diagnoses, such as general anxiety disorder, panic disorder, obsessive-compulsive disorder, posttraumatic stress disorder, major depressive disorder, and so on; (3) a history of psychoactive substance abuse; (4) presence of severe physical diseases such as cardiovascular diseases; (5) pregnancy or lactation.

This study was approved by the Ethics Committee of Second Xiangya Hospital of Central South University. All subjects were informed and fully understood the study and each subject provided a written informed consent.

### Materials

Demographic data were collected from all participants. The duration of illness of BD patients was collected by using the M.I.N.I. Clinical scales, including the Young Mania Rating Scale (YMRS) [27], 17-item Hamilton Depression Rating Scale (HDRS-17) [28], and 14-item Hamilton Anxiety Scale (HAMA-14) [29] were used to assess the manic, depressive, and anxiety symptoms of BD patients, respectively (based on the performance of patients over the past seven days). The subjects in the HC group were carefully screened with M.I.N.I. for DSM-5 to exclude the presence of psychiatric diseases.

Athens Insomnia Scale (AIS) is an internationally recognized self-measurement of sleep quality. It is simple, concise and easy to use, with a total of 8 items, each divided into four levels: 0, 1, 2, and 3, indicating from none to severe [30]. The total score of AIS ranges from 0 to 24, and a total score greater than 6 suggests the presence of insomnia.

Childhood Trauma Questionnaire (CTQ) is a maltreatment inventory that examines childhood growth experiences before age 16. The Chinese version of CTQ short-form (CTQ-SF) [31] was used in this study. It consists of 3 validity items and 25 clinical items which were divided into five clinical subscales: emotional abuse, physical abuse, sexual abuse, emotional neglect, and physical neglect [32]. Each item is rated from 1 to 5 points, representing never, occasionally, sometimes, often, and always, respectively. All the subscale scores meet the following criteria: emotional abuse < 13 points, physical abuse < 10 points, sexual abuse < 8 points, emotional neglect < 15 points, and physical neglect < 10 points means to be free of any form of childhood trauma. As long as one subscale score does not meet the above criteria, it is considered to exist childhood trauma.

Social support was measured in all participants using the Social Support Rating Scale (SSRS). The SSRS contains 10 selective items, 3 for objective support, 4 for subjective support, and 3 for utility of this support [33]. Higher scale scores represent higher levels of social support. A total score of < 20 is generally considered to receive less social support, 20–30 is considered to have general social support, and 30–40 is considered to have satisfactory social support.

The Repeatable Battery for the Assessment of Neuropsychological Status (RBANS) [34] and the Stroop Color-Word Test (Stroop test) were used to measure cognitive function. The RBANS contains 12 subtests which makes up 5 age-adjusted indexes: immediate memory, visuospatial, language, attention, and delayed memory. The Stroop test contains Stroop word, Stroop color, and Stroop color-word.

Suicidality were assessed through self-report questionnaire. Suicide ideation was assessed with a standard question: “Have you ever had thoughts of committing suicide?” with a binary response option “Yes” and “No”. Suicide plan was assessed with a standard question: “Have you ever made a suicide plan?” with a binary response option “Yes” and “No”. Suicide attempt was evaluated with a standard question: “Have you ever tried committing suicide?” with a binary response option “Yes” and “No”. If participants respond positive to these questions, they would continue to be asked about the method, frequency, and further relevant details on the attempts of suicide. Suicide frequency was recorded with a four-level response option “0 time”, “1 time”, “2 times”, and “3 or more times”.

### Statistical analysis

The Statistical Package for Social Sciences (SPSS) version 25.0 (IBM, Chicago) was used for data analysisand Microsoft Power Point was used for graphing. The data were first checked for normality by one-sample Kolmogorov–Smirnov test and quantile-quantile plot (Q-Q plot). Continuous variables were shown as mean (SD), categorical variables were shown as N (%). Variables with skewed distribution were compared by Kruskal-Wallis H and normally distributed variables were compared by One way ANOVA among three groups. Categorical variables were compared by chi-square tests. Two sample *t*-tests were used to compare normally distributed variables between drug-naïve BD and BD with long-term medication groups. The statistically significant level was set to *p* < 0.05 (two-tailed). Post-hoc tests were Bonferroni corrected.

Partial correlation analysis was conducted to analyze the pairwise correlations among childhood trauma, clinical symptoms, and social support to control for education and duration of illness in BD patients. Significant variables were included in Binomial Logistic Regression Analysis (Input) to explore the predictors of suicide ideation, suicide plan, and suicide attempt, and included in Ordinal Logistic Regression Analysis (Input) to explore the predictors of suicide frequency. Multi-collinearity was checked to rule out any association between independent variables.

Bootstrapped Mediation analyses were performed using PROCESS version 3.4 procedure in SPSS (www.afhayes.com) [35]. Total CTQ was entered as a predictor (X), total scale scores of symptoms (HDRS, HAMA, YMRS, and AIS) were entered as outcome variables (Y), and total SSRS was entered as a mediating variable (M) in the mediation model. Total CTQ predicts total SSRS via a path represented by *path a*. Total SSRS predicts clinical symptoms via a path represented by *path b*. The mediation effect (indirect effect) is the cross product of *a* and *b*. The direct effect (path *c’*) represents the relationship between total CTQ and clinical symptoms after controlling for total SSRS in the model. The total effect is the effect of total CTQ on the clinical symptoms without the influence of total SSRS. The number of bootstrap samples was 5000, and years of education were served as covariates in the analysis.

## Results

### Demographic and clinical characteristics

Table 1 shows that there was no difference in age and age of onset among three groups. Both drug-naïve BD and BD with long-term medication group had lower years of education than HC group. Comparing to the drug-naïve BD group, BD with long-term medication group showed lower scores of HDRS, HAMA, and YMRS scores.

**Table 1:** Demographic and clinical data of all participants

	1. Drug naïve BD (*n* = 57)	2. BD with long-term medication (*n* = 64)	3. HCs (*n* = 50)	F/t/H/χ^2^	p	Post-hoc
Age (year)	22.68 (5.12)	24.11 (6.60)	24.24 (2.88)	1.527	0.220^a^	-
Age of onset	19.89(5.51)	18.73(4.70)	-	1.250	0.214 ^b^	-
Education (year)	14.14 (2.25)	14.00 (2.72)	16.12 (1.73)	14.045	<0.001^a^	1, 2<3
Family history of mental illness (n, %)	17 (29.82%)	23 (35.94%)	0	0.509	0.476 ^d^	
Duration of illness	2.29 (2.79)	5.37 (3.99)	-	4.972	<0.001^b^	-
HDRS	22.51 (6.08)	13.17 (8.28)	-	7.122	<0.001^b^	-
HAMA	28.04 (7.09)	16.34 (10.77)	-	7.121	<0.001^b^	-
YMRS	14.21 (7.37)	9.98 (6.11)	-	3.446	0.001^b^	-
AIS	11.54 (5.03)	7.08 (3.67)	4.24 (2.93)	45.861	<0.001^a^	1>2>3
**Childhood trauma**						
Emotional abuse	12.51 (4.70)	9.88 (4.45)	5.88 (1.29)	66.018	<0.001^c^	1>2>3
Physical abuse	7.81 (4.65)	6.22 (2.72)	5.22 (0.79)	25.075	<0.001^c^	1>2>3
Sexual abuse	6.84 (3.06)	6.17 (2.72)	5.12 (0.39)	17.275	<0.001^c^	1, 2>3
Emotional neglect	16.49 (5.44)	14.98 (5.53)	9.74 (4.67)	23.881	<0.001^a^	1, 2>3
Physical neglect	11.04 (4.51)	9.80 (3.82)	7.57 (1.87)	12.510	<0.001^a^	1, 2>3
CTQ total	54.68 (17.05)	47.05 (14.51)	33.50 (7.02)	31.965	<0.001^a^	1>2>3
**Social support**						
Objective support	6.61 (2.44)	7.89 (2.62)	8.66 (2.32)	20.895	<0.001^c^	1<2, 3
Subjective support	13.51 (3.81)	16.98 (4.12)	20.04 (3.94)	36.419	<0.001^a^	1<2<3
Degree for support utilization	6.28 (2.10)	7.14 (1.67)	8.74 (1.70)	24.542	<0.001^a^	1<2<3
SSRS total	26.46 (6.56)	32.02 (6.48)	37.44 (6.07)	39.441	<0.001^a^	1<2<3
**Suicide**						
Ideation (n, %)	55 (96.49%)	56 (87.50%)	13 (26.00%)	77.929	<0.001^d^	1, 2>3
Plan (n, %)	34 (59.65%)	36 (56.25%)	0	49.829	<0.001^d^	1, 2>3
Attempt (n, %)	44 (77.19%)	48 (75.00%)	4 (8.00%)	66.565	<0.001^d^	1, 2>3
Frequency (n, %)				72.740	<0.001^d^	
0	11 (19.30%)	17 (26.56%)	45 (90.00%)	-	-	1, 2>3
1	5 (8.77%)	11 (17.18%)	4 (8.00%)	-	-	-
2	15 (26.32%)	18 (28.13%)	0	-	-	1, 2>3
3 or more	26 (45.61%)	18 (28.13%)	1 (2.00%)	-	-	1, 2>3

(a) One-way ANOVA; (b) Two sample *t* test; (c) Kruskal-Wallis H; (d) Chi-square test

*Post-hoc:* Bonferroni corrected. BD, Bipolar disorder; HC, healthy control; HDRS, Hamilton Depression Rating Scale; HAMA, Hamilton Anxiety Scale; YMRS, Young Mania Rating Scale; AIS, Athens Insomnia Scale; CTQ, Childhood Trauma Questionnaire; SSRS, Social Support Rating Scale

For AIS, emotional abuse, physical abuse, and CTQ total among the three groups, the drug-naïve BD group showed the highest scores, BD with long-term medication group showed the second scores and the HCs group showed the lowest scores. For objective support, the drug-naïve BD group showed lower scores than the BD with long-term medication group and the HCs group. For subjective support, degree for support utilization, and SSRS total, the drug-naïve BD group showed the lowest scores, BD with long-term medication group showed the second scores and the HCs group showed the highest scores.

Comparing to the HCs group, the drug-naïve BD group and the BD with long-term medication group showed higher proportions of suicide ideation, suicide plan, suicide attempt, and suicide frequency. Comparing to cognition of the HCs group, the drug-naïve BD group, and the BD with long-term medication group showed lower scores in immediate memory, visuospatial, language, attention, delayed memory, RBANS total, Stroop word, Stroop color, Stroop color-word, and Stroop total scores (Table 2).

**Table 2:** Cognitive function in all participants

	1. Drug naïve BD (*n* = 57)	2. BD with long-term medication (*n* = 64)	3. HCs (*n* = 50)	F	p	Post-hoc
Immediate memory	39.41 (9.11)	38.16 (9.43)	48.60 (4.75)	25.748	<0.001	1, 2<3
Visuospatial	33.64 (4.02)	34.11 (2.90)	36.78 (1.95)	15.669	<0.001	1, 2<3
Language	28.259 (4.620)	27.555 (5.33)	33.76 (4.64)		<0.001	1, 2<3
Attention	70.63 (10.24)	69.48 (9.20)	78.40 (6.66)	15.847	<0.001	1, 2<3
Delayed memory	47.27 (6.55)	46.59 (8.34)	54.28 (3.76)	21.699	<0.001	1, 2<3
RBANS total	219.21 (26.08)	215.90 (25.79)	251.82 (14.10)	39.427	<0.001	1, 2<3
Stroop word	100.80 (16.99)	94.70 (17.74)	121.22 (16.24)	35.854	<0.001	1, 2<3
Stroop color	69.41 (15.97)	65.42 (15.04)	85.10 (11.44)	28.116	<0.001	1, 2<3
Stroop color-word	38.79 (10.98)	37.97 (9.55)	50.24 (9.22)	25.283	<0.001	1, 2<3
Stroop total	209.00 (38.86)	198.09 (36.77)	256.56 (28.29)	41.905	<0.001	1, 2<3

BD, bipolar disorder; RBANS: Repeatable Battery for the Assessment of Neuropsychological Status

### Associations among childhood trauma, social support, clinical symptoms, cognitive function and suicidality

In the drug-naïve BD group, after controlling for years of education and duration of illness, CTQ and SSRS total scores were correlated with each other, and both CTQ and SSRS total scores were correlated with HDRS, HAMA, and AIS scores (*p* < 0.01, shown in Table 3). However, correlations between suicidality and CTQ/SSRS total scores were not found.

**Table 3:** Correlation of symptoms, childhood trauma, and social support in drug naïve BD patients

*n* = 57	1	2	3	4	5	6
	r					
1. HDRS	1	-	-	-	-	-
2. HAMA	0.744**	1	-	-	-	-
3. YMRS	0.006	0.196	1	-	-	-
4. AIS	0.602**	0.570**	0.161	1	-	-
5. CTQ total	0.401*	0.508**	0.134	0.427*	1	-
6. SSRS total	−0.353*	−0.355*	0.036	−0.378*	−0.341*	1

** *p*<0.001; * *p*<0.05; All correlation coefficients were controlled for years of education and duration of illness

BD, Bipolar disorder; HDRS, Hamilton Depression Rating Scale; HAMA, Hamilton Anxiety Scale; YMRS, Young Mania Rating Scale; AIS, Athens Insomnia Scale; CTQ, Childhood Trauma Questionnaire; SSRS, Social Support Rating Scale

In the BD with long-term medication group, after controlling for years of education and duration of illness, no correlation between CTQ and SSRS total scores was found. Suicide plan was correlated with age, years of education, HAMA, YMRS, and SSRS total scores. Suicide attempt was correlated with age, years of education, YMRS, and CTQ total scores. Suicide frequency was correlated with age, years of education, duration of illness, HAMA, YMRS, AIS, CTQ total, SSRS total scores, and delayed memory (Table 4).

**Table 4:** Correlations of childhood trauma, social support, clinical symptoms, and suicidality in BD patients with long-term medication

*n* = 64	Suicide ideation	Suicide plan	Suicide attempt	Suicide frequency
	r			
Age	−0.237	−0.352**	−0.285*	−0.372**
Education	−0.242	−0.300*	−0.311*	−0.363**
Duration of illness	−0.180	−0.146	−0.157	−0.328**
HDRS	0.004	0.092	0.011	0.177
HAMA	0.210	0.272*	0.231	0.331*
YMRS	0.160	0.328**	0.277*	0.297*
AIS	0.242	0.168	0.159	0.261*
CTQ total	0.046	0.201	0.263*	0.343**
SSRS total	−0.099	0.321**	−0.173	0.296*
Delayed memory	0.172	0.189	0.160	0.349**

Spearman correlation analysis; All correlation coefficients were controlled for years of education and duration of illness; ** *p*<0.01; * *p*<0.05

### Mediation of social support on the association between childhood trauma and clinical symptoms

**Fig. 1:**
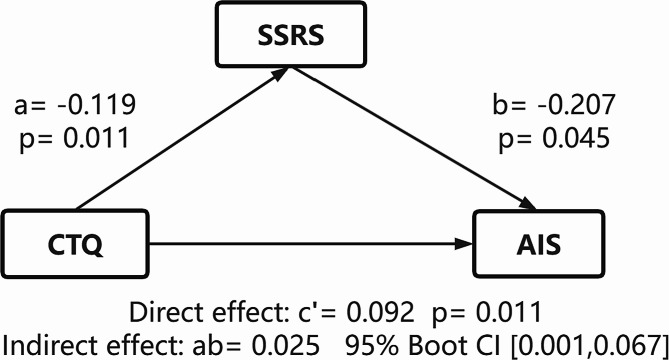
Mediation effect of social support dimensions on the correlation between childhood trauma and Insomnia in drug-naïve BD patients. Years of education and duration of illness were served as covariates. BD, Bipolar Disorder; CTQ, Childhood Trauma Questionnaire; SSRS, Social Support Rating Scale; AIS, Athens Insomnia Scale

The mediation of social support on the association between childhood trauma and AIS scores after controlling for years of education and duration of illness was significant in the drug-naïve BD group (Fig. 1). The results revealed a significant positive indirect effect of CTQ total score on AIS via SSRS total score (standardized indirect effect on AIS ab = 0.025, 95% bootstrap CI 0.001 to 0.067). The mediation of social support on the association between childhood trauma and other clinical symptoms was not significant in the drug-naïve BD group.

The mediation of social support on the association between childhood trauma and clinical symptoms was not significant in the BD with long-term medication group.

### Predictors of suicidality in BD patients with long-term medication

In the BD with long-term medication group, suicide plan and suicide attempt were correlated with age (suicide plan: *r*= -0.352, *p* = 0.004; suicide attempt: *r=* -0.285, *p =* 0.022) and years of education (suicide plan: *r*= -0.300, *p* = 0.016; suicide attempt: *r=* -0.311, *p =* 0.012), respectively. And suicide frequency was correlated with age (*r=* -0.372, *p =* 0.003), education (*r=* -0.363, *p =* 0.003), and duration of illness (*r*= -0.328, *p =* 0.008). Therefore, age and years of education were included in the Binomial Logistic Regression Analysis, and age, years of education, and duration of illness were included in the Ordinal Regression Analysis.

YMRS was a risk factor for suicide plan, and CTQ was a risk factor for suicide attempt. For suicide frequency, years of education was a protective factor, whereas CTQ and delayed memory were risk factors (Table 5).

**Table 5:** Predictors of suicidality using ordinal logistic regression in BD patients with long-term medication

	OR (95% CI)	p
**Suicide plan**		
Age	0.916 (0.818, 1.025)	0.126
Education	0.970 (0.762, 1.237)	0.808
HAMA	0.980 (0.917, 1.047)	0.548
YMRS	1.127 (1.012, 1.256)	0.030*
SSRS total	0.941 (0.851, 1.039)	0.229
**Suicide attempt**		
Age	0.903 (0.799, 1.021)	0.103
Education	0.787 (0.570, 1.085)	0.144
YMRS	1.067 (0.947, 1.202)	0.289
CTQ total	1.088 (1.014, 1.167)	0.018*
**Suicide frequency**		
Age	0.956 (0.851, 1.074)	0.448
Education	0.773 (0.615, 0.973)	0.028*
Duration of illness	0.960 (0.791, 1.165)	0.681
HAMA	1.008 (0.942, 1.080)	0.814
YMRS	1.064 (0.968, 1.169)	0.200
AIS	0.916 (0.753, 1.115)	0.382
CTQ total	1.059 (1.014, 1.104)	0.009*
SSRS total	0.957 (0.871, 1.050)	0.353
Delayed memory	1.091 (1.016, 1.172)	0.016*

* *p*<0.05

## Discussion

We found high levels of childhood trauma, high proportions of suicidality, low social support, and cognitive deficits in drug-naïve and long-term medication female BD patients. Compared to drug-naïve BD patients, long-term medication BD patients showed higher social support and lower childhood trauma, but cognitive deficits and proportions of suicidality were similar. Furthermore, we built two different models in drug-naïve and long-term medication BD patients, respectively. In the drug-naïve BD patients, social support served as a mediator in the relationship between childhood trauma and AIS. In the long-term medication BD patients, insomnia symptoms, childhood trauma, and cognition served as the predictors of suicidality.

### Social support as a mediator between childhood trauma and clinical symptom

Traumatic life events are risk factors for BD, and childhood trauma can exert a lasting effect on mental health [36]. As a stressor, childhood trauma may participate in the pathogenesis of BD via the following mechanisms: (1) altering the developmental progression of the brain; (2) systemic inflammation; (3) alterations in hypothalamic-pituitary-adrenal (HPA) axis function, and disturbance of corticotropin-releasing factor (CRF) neural systems; and (4) genetic influence, such as expression of HPA axis genes, single nucleotide polymorphic gene variants (SNPs) [37].

Social support can be interpreted as a person who is loved, cared for, esteemed, and participates in a network of mutual obligations [38]. The Social Convoy Model provides a conceptual framework that social support provided by parents in early childhood yields a “secure base” and a “safe haven” when faced with stress in subsequent life [39]. And the benefit of social support is prominent in female people [18]. When faced with chronic stress, perceived social support was uniquely related to depression, anxiety, and stress symptoms [40]. The mechanisms by which social support impacts health and well-being are unclear. However, the response to stress was found to be a potential target of social support.

In our study, social support was a mediator between childhood trauma and AIS in the drug-naïve BD patients. A previous study reported that objective support of social support was negatively related with insomnia in major depressive disorder [41]. And it was also reported that a greater degree of childhood trauma was associated with poor sleep health [42]. Our result was consistent with these studies, and we first built this model in drug-naïve BD patients. However, we did not find this model exist in BD patients with long-term medication as previous studies did. In both patients with BD and major depressive disorder, it was also found that social support was a mediator of the relationship between childhood trauma and depression severity [20, 43]. We also did not find this mediation of social support between childhood trauma and depression in our study. These differences could be explained by race, culture, and previous studies excluding the patients with current active suicidal ideation, and age under 18 years old [20].

Treatment strategies targeting social support may serve as effective ways to promote the relief of clinical symptoms and reduce caregiver burden [14]. Mindfulness-based stress reduction (MBSR) program was effective in reducing psychological distress, involving improvement of social support [44]. In addition, online programs which provide web platforms, online support groups and online forums could improve social support by increasing interpersonal contacts and professional communications [45]. One meta-analysis reported that BD patients may benefit from the combination of pharmacotherapy and psychotherapy [46]. By component network meta-analysis, they further found that some specific psychotherapy components delivered in a family or group format was more effective in reducing recurrences than in an individual format [46], suggesting the positive effects of social support. Previous studies have reported that psychosocial treatment could improve total and relationship functioning, life satisfaction, and decrease of time from mood episodes in BD patients [47, 48]. However, current interventions targeting social support are still relatively few, let alone those applied in the field of mental illness.

### Predictors of suicidality

Childhood trauma as a predictor of suicide ideation was previously reported in BD patients [20]. Hippocampus is a brain region which is closely related with cognitive function. Johnston et al. reported that suicide attempters with BD showed significant reductions of gray matter volume in hippocampus when compared to nonattempters with BD [49]. Atrophy of hippocampus is associated with cognitive decline, such as memory [50]. Autobiographical memory, long-term memory and working memory, but not short-term memory, were found to be associated with suicide attempt [51]. In line with these evidences, our results found both childhood trauma and delayed memory could predict suicide frequency but did not find the associated between cognition and suicide attempt in BD patients with long-term medication. BD disease itself, and female patients may explain the differences.

Suicide is regard as a cognitive disorder, and there are some evidences of efficacy of cognitive treatment in suicidality. Self-help mindfulness-based cognitive therapy was effective in reducing suicide ideation of depressive patients, and this effect could last for 3 months [52]. Roberge et al. reported that brief cognitive behavioral therapy could reduce suicide risk by changing cognitive flexibility, which is defined by measures of hopelessness and suicide beliefs. In youth with BD, one study reported that Child- and Family-Focused Cognitive Behavioral Therapy was as effective as psychotherapy treatment-as-usual in reducing likelihood and intensity of suicide ideation[53]. The association between cognition and suicidality found in our study explains why cognitive treatment is effective for suicidality.

There are several limitations in this study. Firstly, this study only included female patients with BD and lacked male patients with BD, which limited the generalization of conclusions to the whole BD population and could not reveal the differences in associations among childhood, social support, cognition, and suicidality between male and female patients with BD. Secondly, the mediation analysis applied in this study predicts a longitudinal process. However, this is a cross-sectional study, and we could not unveil a causal relationship among childhood trauma, social support, and clinical symptoms in BD. The disease itself may also influence social support [15]. Long-term follow-up studies are beneficial to validate the hypothesized model in this study. Thirdly, although we used an age-matched clinical control group, this study is limited by the small sample size. Finally, the assessment of childhood trauma and social support of all participants was based on self-report, which may make the data we obtained somewhat subjective. Moreover, the mood state could influence the retrospective reports of childhood trauma [54]. And we did not evaluate BD in first degree relatives of the patients, which may be a factor that influences the way parents bring up their children. Therefore, there may be some bias in the data used in this study.

## Conclusion

In our study, females with BD showed high levels of childhood trauma and suicidality, as well as low levels of social support and cognitive deficits. The low levels of social support may buffer the negative effect of high levels of childhood trauma on insomnia in BD. Insomnia symptom, childhood trauma, and delayed memory may be predictors of suicide risk in BD patients with medication. 

.

## Data Availability

The datasets used and/or analyzed during the current study are available from the corresponding author on reasonable request.

## Abbreviations

BD: Bipolar disorder
DSM-5: Diagnostic and Statistical Manual of Mental Disorders, Fifth Edition
M.I.N.I: MINI-International Neuropsychiatric Interview Mini diagnostic interview
YMRS: Young Mania Rating Scale
HDRS-17: 17-item Hamilton Depression Rating Scale
HAMA-14: 14-item Hamilton Anxiety Scale
AIS: Athens Insomnia Scale
CTQ: Childhood Trauma Questionnaire
SSRS: Social Support Rating Scale
SPSS: Statistical Package for Social Sciences
HPA: axis hypothalamic-pituitary-adrenal axis
CRF: corticotropin-releasing factor
SNPs: single nucleotide polymorphic gene variants
MBSR: Mindfulness-based stress reduction

## References

[1] Nikolitch K, Saraf G, Solmi M, Kroenke K, Fiedorowicz JG (2023). Fire and darkness: on the Assessment and Management of Bipolar Disorder. Med Clin North Am.

[2] Rhee TG, Gillissie ES, Nierenberg AA, McIntyre RS (2023). Association of current and remitted bipolar disorders with health-related quality of life: findings from a nationally representative sample in the US. J Affect Disord.

[3] Van Rheenen TE, Rossell SL (2014). Objective and subjective psychosocial functioning in bipolar disorder: an investigation of the relative importance of neurocognition, social cognition and emotion regulation. J Affect Disord.

[4] O’Donnell L, Helmuth M, Williams S, McInnis MG, Ryan KA (2023). Predictors of employment status and stability in bipolar disorder: findings from an 8-year longitudinal study. J Affect Disord.

[5] Kanner AM, Saporta AS, Kim DH, Barry JJ, Altalib H, Omotola H, Jette N, O’Brien TJ, Nadkarni S, Winawer MR et al. Mood and Anxiety Disorders and Suicidality in Patients With Newly Diagnosed Focal Epilepsy: An Analysis of a Complex Comorbidity. *Neurology* 2022. 10.1212/WNL.0000000000201671 PMC10074468 36539302

[6] Parial S (2015). Bipolar disorder in women. Indian J Psychiatry.

[7] Zhao H, Zhang X, Xiu M, Wu F (2023). Sex-related differences in parental rearing patterns in young adults with bipolar disorder. Sci Rep.

[8] Mahmoodkhani M, Ghasemi M, Derafshpour L, Amini M, Mehranfard N (2022). Developmental effects of early-life stress on dopamine D2 receptor and proteins involved in noncanonical D2 dopamine receptor signaling pathway in the prefrontal cortex of male rats. J Complement Integr Med.

[9] Wrobel AL, Köhler-Forsberg O, Sylvia LG, Russell SE, Dean OM, Cotton SM, Thase M, Calabrese JR, Deckersbach T, Tohen M (2022). Childhood trauma and treatment outcomes during mood-stabilising treatment with lithium or quetiapine among outpatients with bipolar disorder. Acta Psychiatr Scand.

[10] Zovetti N, Perlini C, Brambilla P, Bellani M (2022). Childhood adversities and bipolar disorder: a neuroimaging focus. Epidemiol Psychiatr Sci.

[11] Wrobel AL, Russell SE, Jayasinghe A, Lotfaliany M, Turner A, Dean OM, Cotton SM, Diaz-Byrd C, Yocum AK, Duval ER (2022). Attachment insecurity partially mediates the relationship between childhood trauma and depression severity in bipolar disorder. Acta Psychiatr Scand.

[12] Agnew-Blais J, Danese A (2016). Childhood maltreatment and unfavourable clinical outcomes in bipolar disorder: a systematic review and meta-analysis. Lancet Psychiatry.

[13] Colic L, Clark A, Sankar A, Rathi DJ, Goldman DA, Kim JA, Villa LM, Edmiston EK, Lippard ETC, Pittman B (2022). Gender-related association among childhood maltreatment, brain structure and clinical features in bipolar disorder. Eur Neuropsychopharmacol.

[14] Shokrgozar S, Rouzbehan V, Zare R, Abdollahi E (2022). Evaluation of patient social support, caregiver burden, and their relationship with the course of the disease in patients with bipolar disorder. Int J Soc Psychiatry.

[15] Stewart RA, Patel TA, McDermott KA, Cougle JR (2022). Functional and structural social support in DSM-5 mood and anxiety disorders: a population-based study. J Affect Disord.

[16] Kendler KS, Myers J, Prescott CA (2005). Sex differences in the relationship between social support and risk for major depression: a longitudinal study of opposite-sex twin pairs. Am J Psychiatry.

[17] Taylor SE, Klein LC, Lewis BP, Gruenewald TL, Gurung RA, Updegraff JA (2000). Biobehavioral responses to stress in females: tend-and-befriend, not fight-or-flight. Psychol Rev.

[18] Bedrov A, Gable SL (2023). Thriving together: the benefits of women’s social ties for physical, psychological and relationship health. Philos Trans R Soc Lond B Biol Sci.

[19] Ning X, Zhang Y, Wang W, Yan H (2022). The association between social support and depression among patients with vitiligo in China. Front Psychol.

[20] Rowe AL, Perich T, Meade T. Childhood cumulative trauma, social support and stress as predictors of illness outcomes and quality of life in bipolar disorder. Aust N Z J Psychiatry 2023:48674231209225. 10.1177/00048674231209225 PMC10960312 37941361

[21] Cardoso T, Bauer IE, Meyer TD, Kapczinski F, Soares JC (2015). Neuroprogression and cognitive functioning in bipolar disorder: a systematic review. Curr Psychiatry Rep.

[22] Zhang L, Lucassen PJ, Salta E, Verhaert P, Swaab DF (2022). Hippocampal neuropathology in suicide: gaps in our knowledge and opportunities for a breakthrough. Neurosci Biobehav Rev.

[23] Gilbert AM, Garno JL, Braga RJ, Shaya Y, Goldberg TE, Malhotra AK, Burdick KE (2011). Clinical and cognitive correlates of suicide attempts in bipolar disorder: is suicide predictable?. J Clin Psychiatry.

[24] Xu X, Xiang H, Qiu Y, Teng Z, Li S, Huang J, Chen J, Tang H, Jin K, Jiang L (2021). Sex differences in cognitive function of first-diagnosed and drug-naïve patients with bipolar disorder. J Affect Disord.

[25] Nierenberg AA, Agustini B, Köhler-Forsberg O, Cusin C, Katz D, Sylvia LG, Peters A, Berk M (2023). Diagnosis and treatment of bipolar disorder: a review. JAMA.

[26] Hu FH, Jia YJ, Zhao DY, Fu XL, Zhang WQ, Tang W, Hu SQ, Wu H, Ge MW, Du W (2023). Gender differences in suicide among patients with bipolar disorder: a systematic review and meta-analysis. J Affect Disord.

[27] Young RC, Biggs JT, Ziegler VE, Meyer DA (1978). A rating scale for mania: reliability, validity and sensitivity. Br J Psychiatry.

[28] Hamilton M (1960). A rating scale for depression. J Neurol Neurosurg Psychiatry.

[29] Hamilton M (1959). The assessment of anxiety states by rating. Br J Med Psychol.

[30] Soldatos CR, Dikeos DG, Paparrigopoulos TJ (2000). Athens Insomnia Scale: validation of an instrument based on ICD-10 criteria. J Psychosom Res.

[31] Bernstein DP, Stein JA, Newcomb MD, Walker E, Pogge D, Ahluvalia T, Stokes J, Handelsman L, Medrano M, Desmond D (2003). Development and validation of a brief screening version of the Childhood Trauma Questionnaire. Child Abuse Negl.

[32] He J, Zhong X, Gao Y, Xiong G, Yao S (2019). Psychometric properties of the Chinese version of the Childhood Trauma Questionnaire-Short Form (CTQ-SF) among undergraduates and depressive patients. Child Abuse Negl.

[33] Zhou FL, Zhang WG, Wei YC, Xu KL, Hui LY, Wang XS, Li MZ (2005). Impact of comorbid anxiety and depression on quality of life and cellular immunity changes in patients with digestive tract cancers. World J Gastroenterol.

[34] Randolph C, Tierney MC, Mohr E, Chase TN (1998). The repeatable battery for the Assessment of Neuropsychological Status (RBANS): preliminary clinical validity. J Clin Exp Neuropsychol.

[35] Hayes AF. Introduction to mediation, moderation, and conditional process analysis: a regression-based approach. 3rd ed. The Guilford; 2022.

[36] Zhang L, Ma X, Yu X, Ye M, Li N, Lu S, Wang J. Childhood trauma and psychological distress: a serial mediation model among Chinese adolescents. Int J Environ Res Public Health 2021, 18(13). 10.3390/ijerph18136808 PMC8297141 34202902

[37] Lippard ETC, Nemeroff CB (2022). Going beyond risk factor: Childhood maltreatment and associated modifiable targets to improve life-long outcomes in mood disorders. Pharmacol Biochem Behav.

[38] Cobb S (1976). Presidential Address-1976. Social support as a moderator of life stress. Psychosom Med.

[39] Greenberg S, Rosenblum KL, McInnis MG, Muzik M (2014). The role of social relationships in bipolar disorder: a review. Psychiatry Res.

[40] Oviedo DC, Pinzón MS, Rodríguez-Araña S, Tratner AE, Pauli-Quirós E, Chavarría C, Posada Rodríguez C, Britton GB (2022). Psychosocial response to the COVID-19 pandemic in Panama. Front Public Health.

[41] Zhang N, Ma S, Wang P, Yao L, Kang L, Wang W, Nie Z, Chen M, Ma C, Liu Z (2023). Psychosocial factors of insomnia in depression: a network approach. BMC Psychiatry.

[42] Brindle RC, Cribbet MR, Samuelsson LB, Gao C, Frank E, Krafty RT, Thayer JF, Buysse DJ, Hall MH (2018). The relationship between Childhood Trauma and Poor Sleep Health in Adulthood. Psychosom Med.

[43] Struck N, Krug A, Feldmann M, Yuksel D, Stein F, Schmitt S, Meller T, Brosch K, Dannlowski U, Meinert S (2020). Attachment and social support mediate the association between childhood maltreatment and depressive symptoms. J Affect Disord.

[44] Tian X, Liao Z, Yi L, Tang L, Chen G, Jiménez Herrera MF (2023). Efficacy and mechanisms of 4-week MBSR on psychological distress in lung cancer patients: a single-center, single-blind, longitudinal, randomized controlled trial. Asia Pac J Oncol Nurs.

[45] Sitges-Maciá E, Bonete-López B, Sánchez-Cabaco A, Oltra-Cucarella J. Effects of e-Health Training and Social Support interventions for Informal caregivers of people with Dementia-A narrative review. Int J Environ Res Public Health 2021, 18(15). 10.3390/ijerph18157728 PMC8345641 34360020

[46] Miklowitz DJ, Efthimiou O, Furukawa TA, Scott J, McLaren R, Geddes JR, Cipriani A. Adjunctive psychotherapy for bipolar disorder: a systematic review and Component Network Meta-analysis. JAMA Psychiatry, 78(2):141–50. 10.1001/jamapsychiatry.2020.2993 PMC7557716 33052390

[47] Swartz HA, Levenson JC, Frank E (2012). Psychotherapy for bipolar II disorder: the role of interpersonal and social rhythm therapy. Prof Psychol Res Pr.

[48] Miklowitz DJ, Otto MW, Frank E, Reilly-Harrington NA, Kogan JN, Sachs GS, Thase ME, Calabrese JR, Marangell LB, Ostacher MJ (2007). Intensive psychosocial intervention enhances functioning in patients with bipolar depression: results from a 9-month randomized controlled trial. Am J Psychiatry.

[49] Johnston JAY, Wang F, Liu J, Blond BN, Wallace A, Liu J, Spencer L, Cox Lippard ET, Purves KL, Landeros-Weisenberger A (2017). Multimodal Neuroimaging of Frontolimbic structure and function Associated with suicide attempts in adolescents and young adults with bipolar disorder. Am J Psychiatry.

[50] Doran S, Carey D, Knight S, Meaney JF, Kenny RA, De Looze C (2023). Relationship between hippocampal subfield volumes and cognitive decline in healthy subjects. Front Aging Neurosci.

[51] Richard-Devantoy S, Berlim MT, Jollant F (2015). Suicidal behaviour and memory: a systematic review and meta-analysis. World J Biol Psychiatry.

[52] Mo Y, Lei Z, Chen M, Deng H, Liang R, Yu M, Huang H (2023). Effects of self-help mindfulness-based cognitive therapy on mindfulness, symptom change, and suicidal ideation in patients with depression: a randomized controlled study. Front Psychol.

[53] Weinstein SM, Cruz RA, Isaia AR, Peters AT, West AE (2018). Child- and Family-focused cognitive behavioral therapy for Pediatric Bipolar disorder: applications for suicide Prevention. Suicide Life Threat Behav.

[54] Carnelley KB, Pietromonacó PR, Jaffe K (1994). Depression, working models of others, and relationship functioning. J Pers Soc Psychol.

